# Specificity of Dengue NS1 Antigen in Differential Diagnosis of Dengue and Zika Virus Infection

**DOI:** 10.3201/eid2209.160725

**Published:** 2016-09

**Authors:** Séverine Matheus, Rachida Boukhari, Bhety Labeau, Valérie Ernault, Laetitia Bremand, Mirdad Kazanji, Dominique Rousset

**Affiliations:** Institut Pasteur de la Guyane, Cayenne, French Guiana (S. Matheus, B. Labeau, L. Bremand, M. Kazanji, D. Rousset);; Centre Hospitalier de l’Ouest-Guyanais, Saint-Laurent du Maroni, French Guiana (R. Boukhari, V. Ernault)

**Keywords:** Dengue, Zika, cross-reactivity, nonstructural protein, NS1 protein, NS1 antigen, differential diagnosis, viruses, specificity

**To the Editor:** Circulation of new arboviruses of the genus *Flavivirus* poses a major problem for differential diagnosis. Zika virus, a mosquitoborne virus of the family *Flaviviridae*, is closely related to other arboviruses circulating in the Americas, including dengue, yellow fever, Saint Louis encephalitis, and West Nile viruses (*1*,*2*). Serologic cross-reactivity between these arboviruses is common; thus, to ensure optimal patient care and accurate epidemiologic surveillance, an effective differential diagnosis is required in regions with active transmission of dengue virus and circulation of Zika virus (*2*–*4*).

Cross-reactivity between flaviviruses has been reported in antibody assays and in tests for Dengue nonstructural 1 glycoprotein (NS1) antigen. Gyurech et al. (*5*) reported false-positive test results for dengue NS1 antigen in a patient with acute Zika virus infection. Of the 3 NS1 tests used in that study, only the SD Bioline Dengue Duo (Standard Diagnostics, Inc., Gyeonggi-do, South Korea) showed positive results for 3 of 4 sequential serum samples from the patient.

Cross-reactivity in NS1 dengue tests (ELISA and immunochromatographic) using serum samples from patients with acute Zika virus infection would have medically significant consequences. We therefore conducted a retrospective analysis of the differential diagnosis for dengue and Zika virus infections since the beginning of the Zika virus outbreak in French Guiana, a department of France on the northeast coast of South America.

French Guiana is subject to endemoepidemic circulation of dengue and experienced a large outbreak of chikungunya in 2014. We conducted our study from December 17, 2015 (the time of biologic confirmation of the first case of Zika virus disease in French Guiana), through March 2, 2016. During that time, the incidence of dengue virus infection in French Guiana was low, and only 1 sporadic case was confirmed. We studied clinical samples collected during this period from all patients with suspected arbovirus infection. Samples were analyzed for the differential diagnosis of dengue, chikungunya, and Zika virus disease. Zika virus diagnosis was conducted by the National Reference Centre for Arboviruses (NRC) at the Institut Pasteur of French Guiana, in Cayenne, according to the real-time reverse transcription PCR (rRT-PCR) protocol described by Lanciotti et al. (*3*). Dengue diagnosis was routinely performed by all medical diagnostic laboratories using various rRT-PCR techniques or dengue NS1 test kits. We used the same SD Bioline Dengue Duo test used by Gyurech et al. (*5*); this test was performed at the laboratory of the Centre Hospitalier de l’Ouest-Guyanais in Saint-Laurent du Maroni, French Guiana. We also used the Platelia Dengue NS1 Ag kit (Bio-Rad, Marnes-la-Coquette, France); the assay was performed at NRC.

Since Zika virus first appeared in French Guiana, the NRC has investigated 270 samples collected 0–5 days after fever onset for molecular diagnosis of Zika virus and dengue NS1. Of the 270 suspected patients, 65 were confirmed positive for acute Zika virus infection by rRT-PCR of serum, urine, or both. The mean cycle thresholds (± SDs) were 33.0 (± 3.4) for serum samples and 33.2 (± 3.5) for urine samples (Table). Of the 65 acute-phase Zika virus–positive serum samples, 36 were also tested with the Platelia Dengue NS1 test, 21 were tested with the SD Bioline Dengue Duo test, and 8 were tested with both tests; none of the results were positive. Of the 205 Zika virus–negative samples, 204 were also negative for dengue NS1; only 1 patient had a positive dengue NS1 test result, and the infection was confirmed by molecular investigations to be a case of acute dengue-1 disease.

**Table:** RT-PCR results for 65 persons with clinical samples tested during the acute phase of Zika virus infection, French Guiana, December 17, 2015–March 2, 2016*

Days from symptom onset to sample collection	No. cases	RT-PCR results, by clinical sample							
		Serum only			Urine only			Serum and urine	
		No. samples	C_t_, mean ± SD		No. samples	C_t_, mean ± SD		No. samples	C_t_, mean ± SD
0	8	5	31.8 ± 2.8		1	34.6†		2	Serum: 28.6 ± 10.45; urine: 28.9 ± 11.3
1	12	4	29.9 ± 4.6		2	36.5 ± 0.3		6	Serum: 36.0 ± 1.7; urine: 33.6 ± 4.4
2	20	13	32.8 ± 3.0		1	33.7†		6	Serum: 34.2 ± 3.6; urine: 33.0 ± 1.4
3	15	8	33.6 ± 3.6		2	34.2 ± 1.5		5	Serum:33.6 ± 2.8; urine: 34.1 ± 1.6
4	7	5	31.7 ± 2.4		1	28.9†		1	Serum:32†; urine: 32†
5	3	0	NA		1	31.7†		2	Serum: 35.1 ± 0.8; urine: 32.9 ± 3.9
Total	65	35	32.5 ± 3.1		8	33.8 ± 2.6		22	Serum: 33.6 ± 3.8; urine: 32.9 ± 3.8

*C_t_, cycle threshold; NA, not applicable; RT-PCR, reverse transcription PCR. †SD not determined.

This retrospective analysis of dengue and Zika virus diagnoses indicates that no false-positive dengue NS1 test results occurred among samples with acute-phase Zika virus infection. Indeed, samples from all 65 patients with rRT-PCR–confirmed acute Zika virus infection were negative by both dengue NS1 tests. Zika virus is closely related to dengue virus, and during the acute phase of disease, Zika virus might release NS1 into patients’ serum; however, this putatively released nonstructural protein does not appear to cross-react with the dengue NS1 tests used in our study. No Zika NS1 antigen assay currently exists, and acute-phase release of Zika NS1 has not been verified. If a Zika virus NS1 test is developed, it should be evaluated for cross-reactivity with serum from patients with acute dengue infection. The false-positive result reported by Gyurech et al. (*5*) for dengue NS1 antigen in a patient with acute Zika virus infection requires further investigation. Little is known about false-positive NS1 tests. Zika virus might show cross-reactivity with other flaviviruses and possibly cytomegalovirus, and hematologic disorders might cause NS1 positivity (*6*,*7*).

The co-circulation of Zika virus and dengue virus in the Americas is causing a health emergency. Our findings show that dengue NS1 antigen assays are still entirely appropriate for dengue surveillance, even during the epidemic circulation of Zika virus.
